# Editorial Department

**Published:** 1856-03

**Authors:** 


In the libel suit brought by John D. Hill *vs.* Austin Flint and Sanford B. Hunt, as editors of the Buffalo Medical Journal, which has come to trial since our last issue, the jury — (and *such* a jury!) — brought in a verdict of damages to the plaintiff to the amount of $500. We have in preparation a full report of the trial, which we may, and may not, give to our readers next month.

In the meantime we wish to impress upon "all and every" (we are growing legal in our language) of our subscribers, that now is a particularly good time to pay up. Give us the sinews of war, good friends!

